# Supplementary material for: Research on a virtual-real fusion experimental system for the cutting part of a boom-type roadheader
Source: PLoS One. 2025 Aug 18;20(8):e0330291. doi: 10.1371/journal.pone.0330291 (PMC12360573; doi:10.1371/journal.pone.0330291)
Supplement: Attachment — Submitted filename: response letter.doc [file pone.0330291.s006.doc]

**List of Responses**

Dear editors and reviewers:

We have carefully studied your valuable opinions on our manuscript "Research on virtual-real fusion experiment system of the cutting part of the boom-type roadheader". Thank you for giving us the opportunity to make revisions.

We highly value your insightful comments on the previous version. In response, we have carefully processed each comment as follows. All the modified parts are highlighted in the original draft. We sincerely hope that our revisions meet your expectations.

**Reviewer 1:**

**Question 1:** In the process of constructing the rectangular coal-rock unit in Section 2.1 of the paper to form the input matrix of the digital mapping of the pick load, the author considered parameters such as the depth, angle, and speed of the pick cutting into the coal-rock, as well as the damage degree of the cut coal-rock unit. However, the author did not elaborate in detail on the reasons for selecting these parameters. It is recommended that the author supplement relevant content and explain in detail the necessity and rationality of selecting these parameters.

**Response 1:** Thank you for your valuable suggestions.

To obtain the external load conditions of the boom-type roadheader and simulate the mechanical state of the boom-type roadheader, it is necessary to obtain the mechanical state of the cutting teeth. The acquisition of the mechanical state of the cutting tooth is achieved through the mapping relationship $f_{io}\text{：}\left[i\right]\to\left[o\right]$ of the coupling information from coal-rock to the cutting tooth. Among them, $\left[i\right]$ is the parameter that affects the mechanical state of the cutting tooth, and $\left[o\right]$ is the mechanical state information of the cutting tooth. During the process of cutting coal-rock by the cutting tooth, in addition to the mechanical properties of the coal-rock materials, the factors affecting the mechanical state of the cutting tooth mainly include the contact area and contact pressure between the coal-rock and the cutting tooth. Among them, the contact area between the coal-rock and the cutting tooth is mainly affected by factors such as the depth and angle at which the cutting tooth cuts into the coal-rock, and the contact pressure between the coal-rock and the cutting tooth is mainly affected by factors such as the cutting speed of the cutting tooth and the damage degree of the cutting coal-rock unit. Therefore, in this study, parameters such as the depth, angle, speed at which the cutting tooth cuts into the coal-rock, and the degree of damage to the cut coal-rock unit are taken as parameters in$\left[i\right]$. (The second paragraph of Section 2.1 in the paper has been modified and marked in highlighted font. As shown below)

**Modified part:**

During cutting tooth penetration into coal-rock, beyond the mechanical properties of the coal-rock materials, the mechanical state of the cutting tooth depends primarily on contact area and contact pressure. Specifically: Contact area is governed by the penetration depth and attack angle of the cutting tooth; Contact pressure depends on the cutting speed and damage coefficient of the coal-rock unit. Therefore, in this study, parameters such as the penetration depth, attack angle, cutting speed, damage coefficient are taken as parameters in . As shown in Fig. 2. The penetration depth and attack angle are derived from the homogeneous transformation matrix of the cutting tooth relative to the coal-rock element's local coordinate system:

**Question 2:** In Fig.2 of Section 2.1 of the paper, the coordinate colors are dim, which may affect the readers' understanding of the chart content. It is recommended that the author adjust the coordinate colors to make them more eye-catching and clearly mark the main parameters to enhance the readability of the chart.

**Response 2: Thank you for your valuable suggestions.**

In the text, Fig.2 is redrawn, the color and direction of the coordinate system are modified, and parameter identifiers are added. As shown in the following figure and marked in highlighted font in the paper.

**Modified part:**

**Fig. 2.** Cutting tooth cutting position diagram

**Question 3:** In Section 3.2 of the paper, the Denavit-Hartenberg notation (DH method) is used for the motion equations, but the DH parameters are not clearly given, and there is a lack of a detailed description of the specific way the pose matrix is used in the test system. It is recommended to add relevant explanations so that readers can better understand the relevant content.

**Response 3: Thank you for your valuable suggestions.**

The part of the Modifie-Denavite-Hartenberg (MDH) coordinate system in Section 2.3 has been modified. Parameters and joint variable tables have been added, and the description of the role of the motion equation has been enhanced. (Section 3.2 of the paper has been modified and marked in highlighted font. As shown below.)

**Modified part:**

The kinematic equation of the boom-type roadheader's cutting module are derived using the Modifie-Denavite-Hartenberg (MDH) method, as shown in Fig. 7, with the fixed hinge seat of the moving cylinder serving as the global coordinate origin $O_{0}\left(0,0,0\right)$. The MDH parameters and joint variables of each structure are listed in Table 1. The movement of the cutting part of the boom-type roadheader is controlled by the displacement $x_{1}$ of the moving cylinder, the displacement of the rotational cylinder, the displacement of the swing arm cylinder and the rotation angle of the cutting head. The pose state of the cutting part of the boom-type roadheader is calculated by combining the displacement change data output by the digital mapping body of each cylinder structure.

**Table 1.** The MDH parameters and joint variables of the cutting part of the boom-type roadheader

| Link |  |  |  |  |
| --- | --- | --- | --- | --- |
| 1 | 0 |  | 0 | （variate） |
| 2 | （variate） |  | 0 |  |
| 3 | （variate） |  | 0 | 0 |
| 4 | （variate） |  | 0 |  |

The motion equation of the base is obtained by calculating the displacement of the moving cylinder:

(8)

Here, is the displacement of the moving cylinder, is the pose matrix of the base, is the motion equation of the base,  is the displacement data of the moving cylinder foundation, is the displacement change caused by the passive deformation of the moving cylinder. The motion equation of the rotating platform is obtained by translating the displacement of the moving cylinder and the displacement of the rotating cylinder:

(9)

Here, is the distance between the coordinate of the rotating platform and the coordinate of the base, is the pose matrix of rotating platform, is the motion equation of rotating platform,  is the angle of the rotating platform obtained by combining the displacement data of the rotating cylinder foundation with Equation 7, is the corrected displacement data of the rotating cylinder and the corrected angle of the rotating platform obtained by Equation 7. The motion equation of the swing arm is obtained through the displacement of the moving cylinder, the displacement of the rotating cylinder and the displacement of the swing arm cylinder:

(10)

Here, is the pose matrix of rotating platform, is the motion equation of rotating platform, is the swing arm angle obtained by combining the displacement data of the swing arm cylinder foundation with Equation 7, is the correction angle of the swing arm obtained by correcting the displacement data of the swing arm cylinder and Equation 7. The motion equation of the cutting head is obtained through the displacement of the moving cylinder, the displacement of the rotating cylinder, the displacement of the swing arm cylinder and the rotation angle of the cutting head:

(11)

Here, is the distance between the coordinates of the cutting head and the coordinates of the swing arm, is the pose matrix of the cutting head, is the motion equation of the cutting head. is the rotation angle of the cutting head.

Finally, the kinematic equations of individual structural components are integrated with their respective digital twins, establishing a full-machine kinematic model that simulates the pose dynamics of the boom-type roadheader's cutting module.

**Question 4:** For the acquisition of the pose information and mechanical information of the virtual-real fusion test system, it is necessary to clearly state what types of sensors are used, the specific measurement methods, and the arrangement of the sensors. At the same time, it is also necessary to explain how to use the local and limited sensor data to reflect the mechanical characteristics of the whole machine in different poses.

**Response 4: Thank you for your valuable suggestions.**

The attitude and mechanical states of each structural body comprise sensor-derived data and digital twin outputs. Among them, the sensor data are obtained from the displacement sensor and pressure sensor from the translational cylinder, the displacement sensor and pressure sensor from the rotating cylinder, the displacement sensor and pressure sensor from the swing arm cylinder, and the torque and speed sensor from the cutting head. Non-sensor data primarily consist the mechanical data and pose correction data of each structure, derived via component-level digital twins. To ensure output accuracy, input-output datasets must encompass diverse operating conditions. The input data set should cover all factors affecting outputs, as shown in Fig 5. Numerical simulations under various working conditions are conducted by changing the input parameters to cover as many possible working conditions as possible. Mechanical states and pose corrections extracted from these simulations construct the datasets. Deep learning establishes the mapping between input parameters and output responses, enabling real-time prediction of the boom-type roadheader's mechanical states. This approach achieves real-time numerical simulation capabilities, providing stress and deformation data at grid nodes.. The digital simulation employs the LS-DYNA solver, the solid adopts the Lagrangian, the oil adopts the Arbitrary Lagrangian Eulerian(ALE), and the joint adopts the Kinematic pair connection.（Paragraph 3 of Section 3.1 in the paper has been modified and marked in highlighted font. As shown below:）

**Modified part:**

The attitude and mechanical states of each structural body comprise sensor-derived data and digital twin outputs. Among them, the sensor data are obtained from the displacement sensor and pressure sensor from the translational cylinder, the displacement sensor and pressure sensor from the rotating cylinder, the displacement sensor and pressure sensor from the swing arm cylinder, and the torque and speed sensor from the cutting head. Non-sensor data primarily consist the mechanical data and pose correction data of each structure, derived via component-level digital twins. To ensure output accuracy, input-output datasets must encompass diverse operating conditions. The input data set should cover all factors affecting outputs, as shown in Fig 5. Numerical simulations under various working conditions are conducted by changing the input parameters to cover as many possible working conditions as possible. Mechanical states and pose corrections extracted from these simulations construct the datasets. Deep learning establishes the mapping between input parameters and output responses, enabling real-time prediction of the boom-type roadheader's mechanical states. This approach achieves real-time numerical simulation capabilities, providing stress and deformation data at grid nodes. The digital simulation employs the LS-DYNA solver, the solid adopts the Lagrangian, the oil adopts the Arbitrary Lagrangian Eulerian(ALE), and the joint adopts the Kinematic pair connection.

**Question 5:** Section 5.3 of the paper describes the sources of the environmental state and reward information during the virtual training process and the virtual-real fusion training process. During the virtual training, both the environmental state and reward information come from the calculation data of the virtual test system. In the virtual-real fusion training process, the environmental state information comes from the sensor data, and the reward information comes from the calculation data of the virtual test system. In order to display these data information more clearly, it is recommended that the author present them intuitively in the form of a chart for easy comparative analysis.

**Response 5: Thank you for your valuable suggestions.**

Virtual training conducts deep reinforcement learning within the virtual experiment system, utilizing virtual sensor data and mechanical parameters (e.g., stress/deformation) to provide environmental states and reward values for the agent, with action strategies executed by the digital twin. Virtual-real fusion training simultaneously operates in both virtual and physical systems. Strain gauges installed at key positions of the boom-type roadheader's cutting module collect physical stress data, while the physical system acquires sensor data and the virtual system gathers virtual sensor data and mechanical states. Data from both platforms undergo outlier removal and averaging to supply environmental states and reward values, with agent-derived action strategies executed concurrently by the physical entity and its digital twin. In Section 5.3, the sources of environmental states and reward information during the virtual training and virtual-real fusion training processes were graphically plotted, as shown in the following figure.（Section 5.3 in the paper has been modified and marked in highlighted font. As shown below:）

**Modified part:**

**Fig. 11.** Physical experiment system and virtual experiment system

The training process takes the DDPG algorithm as the core and conducts 300 virtual trainings and 10 virtual-real fusion trainings through the virtual-real fusion experiment system of the cutting part. Virtual training conducts deep reinforcement learning within the virtual experiment system, utilizing virtual sensor data and mechanical parameters (e.g., stress/deformation) to provide environmental states and reward values for the agent, with action strategies executed by the digital twin. Virtual-real fusion training simultaneously operates in both virtual and physical systems. Strain gauges installed at key positions of the boom-type roadheader's cutting module collect physical stress data, while the physical system acquires sensor data and the virtual system gathers virtual sensor data and mechanical states. Data from both platforms undergo outlier removal and averaging to supply environmental states and reward values, with agent-derived action strategies executed concurrently by the physical entity and its digital twin. The cutting task is linear cutting, and the environment state and reward information in the virtual training process are derived from the calculation data of the virtual experiment system. In the process of virtual-real fusion training, the environment state information comes from the sensor data, and the reward information comes from the calculation data of the virtual experiment system. As shown in Fig. 11.

**Question 6:** The number of references cited is relatively small. It is recommended that the author appropriately increase the number of references cited, widely draw on the research achievements in related fields, and further enrich the content of the article.

**Response 6: Thank you for your valuable suggestions.**

The introduction in the paper has been modified and supplemented, and references have been added.（The introduction part and the references part in the paper have been modified and marked in highlighted font. As shown below:）

**Modified part:**

(1) Introduction：

The boom-type roadheader is a tunneling machine designed for rapid excavation in underground coal mine tunnels. Accurate measurement and control of its operational posture, along with the development of intelligent cutting systems and mechanical property optimization, are critical for enhancing tunneling safety and efficiency [1,2]. For instance, intelligent control of rotational and swing speeds during excavation maintains consistent cutting torque and structural stress, thereby minimizing equipment vibration and wear under variable coal-rock conditions [3]. The integration of image processing and deep learning further enhances system intelligence while reducing operational safety risks [4]. Traditional research methods relying on full-scale physical prototypes face limitations including prolonged development cycles, high costs, and challenges in replicating extreme operating conditions. Studies involving pose recognition and adaptive control which depend on sensor data and simulations confront additional hurdles: adverse environmental factors at tunnel faces, difficulties in acquiring real-time coal-rock properties and equipment status data, and stringent safety protocols collectively escalate experimental durations, costs, and risks [5-7]. Overcoming these cost and efficiency barriers remains a pivotal challenge in advancing intelligent roadheader development.

Current methodologies primarily involve physical experiments and numerical simulations. While physical experiments provide reliable data, their implementation involves repetitive tasks, accelerated equipment degradation, and high temporal-economic costs, limiting multi-parameter combinatorial validation. For instance, fabricating coal-rock specimens and deploying sensor networks demand substantial labor and material investments [8]. Data-intensive approaches like deep learning face scalability challenges due to the prohibitive costs of acquiring labeled datasets through full-scale underground or surface cutting experiments [9-11]. Although numerical simulations reduce costs through virtual data generation, two critical limitations persist: (1) accuracy degradation from idealized boundary conditions, and (2) inadequate representation of system-level behaviors when simulating isolated components [12]. System-scale simulations further require complex coupling across multiple software platforms, compounded by the lack of real-time cross-validation between numerical and physical domains [13-16]. This decoupling prevents dynamic error correction, necessitating post-hoc experimental verification of simulation datasets.

Current experimental approaches predominantly employ bench-scale or reduced-dimension prototypes to mitigate the complexity of boom-type roadheader testing. Existing platforms simulate operational states through cutting head pose recognition, control system emulation, and concrete cutting modules. However, material-based simulations (e.g., concrete/gypsum-coal mixtures) lack real-time tunability of mechanical properties, while requiring extensive manual calibration to approximate coal-rock behaviors [17]. Although theoretical modeling and virtual simulations partially alleviate these issues, physical validation remains mandatory [18]. Integrating physical prototypes with theoretical models and virtual simulations into a unified virtual-real testing system could systematically mitigate experimental complexity. Specifically, virtual sensors enable automated data acquisition during testing cycles, circumventing the need for intrusive physical instrumentation. This integration establishes a foundational platform for advanced roadheader research with enhanced self-sensing capabilities.

Digital twin technology represents a prominent research field in virtual-real system integration. This technology enables real-time synchronization between physical entities and their virtual counterparts through bidirectional data exchange [19-21]. Despite these advances, current implementations predominantly focus on pose monitoring and control of boom-type roadheaders, while neglecting integrated mechanical simulation and experimental validation [22-24]. Deep learning exhibits exceptional nonlinear regression capabilities, allowing it to approximate nonlinear interactions between boundary constraints and structural response fields when trained on numerical datasets [25,26]. Such models facilitate the generation of reduced-order simulations and digital twin mappings, thereby accelerating mechanical analysis [27-30]. By coupling physical prototypes with their digital twins, a hybrid experimental framework emerges, unifying virtual and physical testing modalities under a single architecture.

This study aims to develop a cutting module experimental system for boom-type roadheaders, establishing a virtual-dominant physical calibration paradigm to reduce experimental costs, enhance dataset accuracy, and improve system intelligence. The proposed system integrates five core functionalities:

（2）References：

[1] Li Q, Liu S, Gao M. Multiobjective optimization of roadheader shovel-plate parameters using gray weight and particle swarm optimization. IEEE Access. 2022 Sep 26;10:104555-66. <https://doi.org/10.1109/ACCESS.2022.3210165>.

[2] Wang S, Wang D, Ma A, et al. Intelligent Robust Control of Roadheader Based on Disturbance Observer. Actuators 2025, 14(1):36. <https://doi.org/10.3390/act14010036>.

[4] Yang W, Zhang X, Ma H, et al. Geometrically driven underground camera modeling and calibration with coplanarity constraints for a boom-type roadheader. IEEE Transactions on Industrial Electronics. 2020 Aug 25;68(9):8919-29. <https://doi.org/10.1109/TIE.2020.3018072>.

[6] Lin, H, Liu, W, Zhang, D, et al. Study on the degradation mechanism of mechanical properties of red sandstone under static and dynamic loading after different high temperatures. Sci Rep 15, 11611 (2025). <https://doi.org/10.1038/s41598-025-93969-4>.

[7] Wang L, Xue Y, Liu J, et al. Mechanical behavior and fracture mechanism of high-temperature granite cooled with liquid nitrogen for geothermal reservoir applications. Physics of Fluids. 2025 Feb 1;37(2). <https://doi.org/10.1063/5.0253668>.

[17] Dong Z, Zhang X, Yang W, et al. Automatic Cutting Speed Control System of Boom-Type Roadheader Based on Geological Strength Index. Minerals. 2022; 12(12):1582. <https://doi.org/10.3390/min12121582>

[18] Tian, Jie, Suyu Wang, Miao Wu. Kinematic models and simulations for trajectory planning in the cutting of Spatially-Arbitrary crosssections by a robotic roadheader. Tunnelling and Underground Space Technology 78 (2018): 115-123. <https://doi.org/10.1016/j.tust.2018.03.032>.

[24] Cheluszka P, Remiorz E, Rostami J. The Use of a Roadheader Simulator in Research of Dynamics and Energy-Consumption of Excavating Underground Roadways and Tunnels. Energies. 2022; 15(18):6673. <https://doi.org/10.3390/en15186673>.

**Reviewer 2:**

**Question 1:** In the introduction section of the article, only some shortcomings of traditional research methods are listed, with limited comparative analysis of recent achievements by other scholars in the improvement of tunnel boring machine experimental systems or similar applications of virtual-real fusion technology. It is recommended to supplement relevant content to more clearly highlight the unique innovations of this study.

**Response 1: Thank you for your valuable suggestions.**

The introduction in the paper has been modified and supplemented.（The introduction in the paper have been modified and marked in highlighted font. As shown below:）

**Modified part:**

The boom-type roadheader is a tunneling machine designed for rapid excavation in underground coal mine tunnels. Accurate measurement and control of its operational posture, along with the development of intelligent cutting systems and mechanical property optimization, are critical for enhancing tunneling safety and efficiency [1,2]. For instance, intelligent control of rotational and swing speeds during excavation maintains consistent cutting torque and structural stress, thereby minimizing equipment vibration and wear under variable coal-rock conditions [3]. The integration of image processing and deep learning further enhances system intelligence while reducing operational safety risks [4]. Traditional research methods relying on full-scale physical prototypes face limitations including prolonged development cycles, high costs, and challenges in replicating extreme operating conditions. Studies involving pose recognition and adaptive control which depend on sensor data and simulations confront additional hurdles: adverse environmental factors at tunnel faces, difficulties in acquiring real-time coal-rock properties and equipment status data, and stringent safety protocols collectively escalate experimental durations, costs, and risks [5-7]. Overcoming these cost and efficiency barriers remains a pivotal challenge in advancing intelligent roadheader development.

Current methodologies primarily involve physical experiments and numerical simulations. While physical experiments provide reliable data, their implementation involves repetitive tasks, accelerated equipment degradation, and high temporal-economic costs, limiting multi-parameter combinatorial validation. For instance, fabricating coal-rock specimens and deploying sensor networks demand substantial labor and material investments [8]. Data-intensive approaches like deep learning face scalability challenges due to the prohibitive costs of acquiring labeled datasets through full-scale underground or surface cutting experiments [9-11]. Although numerical simulations reduce costs through virtual data generation, two critical limitations persist: (1) accuracy degradation from idealized boundary conditions, and (2) inadequate representation of system-level behaviors when simulating isolated components [12]. System-scale simulations further require complex coupling across multiple software platforms, compounded by the lack of real-time cross-validation between numerical and physical domains [13-16]. This decoupling prevents dynamic error correction, necessitating post-hoc experimental verification of simulation datasets.

Current experimental approaches predominantly employ bench-scale or reduced-dimension prototypes to mitigate the complexity of boom-type roadheader testing. Existing platforms simulate operational states through cutting head pose recognition, control system emulation, and concrete cutting modules. However, material-based simulations (e.g., concrete/gypsum-coal mixtures) lack real-time tunability of mechanical properties, while requiring extensive manual calibration to approximate coal-rock behaviors [17]. Although theoretical modeling and virtual simulations partially alleviate these issues, physical validation remains mandatory [18]. Integrating physical prototypes with theoretical models and virtual simulations into a unified virtual-real testing system could systematically mitigate experimental complexity. Specifically, virtual sensors enable automated data acquisition during testing cycles, circumventing the need for intrusive physical instrumentation. This integration establishes a foundational platform for advanced roadheader research with enhanced self-sensing capabilities.

Digital twin technology represents a prominent research field in virtual-real system integration. This technology enables real-time synchronization between physical entities and their virtual counterparts through bidirectional data exchange [19-21]. Despite these advances, current implementations predominantly focus on pose monitoring and control of boom-type roadheaders, while neglecting integrated mechanical simulation and experimental validation [22-24]. Deep learning exhibits exceptional nonlinear regression capabilities, allowing it to approximate nonlinear interactions between boundary constraints and structural response fields when trained on numerical datasets [25,26]. Such models facilitate the generation of reduced-order simulations and digital twin mappings, thereby accelerating mechanical analysis [27-30]. By coupling physical prototypes with their digital twins, a hybrid experimental framework emerges, unifying virtual and physical testing modalities under a single architecture.

This study aims to develop a cutting module testing system for boom-type roadheaders, establishing a virtual-dominant physical calibration paradigm to reduce experimental costs, enhance dataset accuracy, and improve system intelligence. The proposed system integrates five core functionalities:

**Question 2:** The stress values in the text use incorrect units, such as 100mpa.

**Response 2:** **Thank you for pointing out the mistakes.**

We are very sorry for the errors in the paper. We have corrected the mistakes regarding stress units in the paper and made language modifications and format checks throughout the paper. We sincerely thank you for your strict supervision, which has helped us enhance the professionalism of the paper.

**Question 3:** After optimization, the stress overrun ratio decreases by approximately 10%. To demonstrate the superiority of DDPG, comparisons with other algorithms can be conducted.

**Response 3: Thank you for your valuable suggestions.**

In the paper, a virtual-real fusion testing system for the cutting part of the roadheader was constructed, and the accuracy of the system was verified through the cutting test. To further verify the ability of the system to conduct cutting test research on the cutting section of the roadheader and the virtual-dominated - entity calibration function, taking the application research of the DDPG algorithm in the cutting control process as an example, virtual tests and virtual-real fusion tests were carried out. The optimization effect of the DDPG algorithm is mainly introduced in the article, while the functional verification of the test system is ignored, which causes ambiguity. Thanks to the experts for their opinions. Section 5.3 has now been modified to highlight the functional verification of the virtual-real fusion test system for the cutting part of the roadheader. Since the main research content of this paper is the virtual-real fusion testing system of the cutting part of the roadheader, rather than the optimization of mechanical properties, a DDPG algorithm is adopted to conduct functional verification of the test system. However, other reinforcement learning algorithms will be attempted later to conduct performance optimization experiments on the cutting part of the roadheader.（Section 5.3 in the paper has been modified and marked in highlighted font. As shown below:）

**Modified part:**

**Fig. 13.** Functional proof of the virtual-real fusion experiment system

The results show that adopting the DDPG algorithm for extensive virtual training supplemented by targeted physical validation within the virtual-real fusion system successfully optimized the cutting module's structural stress control in the cutting module (Fig 13). This process validated the system's capability to construct homogeneous/heterogeneous coal-rock digital models through hard coal-rock mechanical simulation. During hard coal-rock cutting simulations, average stress errors below 7.63 MPa in swing arm cylinders and rotary cylinders confirmed accurate identification and fitting of cutting tooth-coal-rock coupling relationships. Real-time monitoring of digital twins and physical entities was achieved through synchronized physical/virtual sensor data acquisition. High-fidelity pose simulation and mechanical data output demonstrated effective real-time motion/environment simulation with whole-machine multi-modal perception. The 10% structural stress reduction validated cross-domain virtual-real data fusion for enhanced experimental dataset accuracy.

**Question 4:** Some latest research work related with this TOPIC can be referred. Study on the degradation mechanism of mechanical properties of red sandstone under static and dynamic loading after different high temperatures.

**Response 4: Thank you for your valuable suggestions.**

In the paper, the input and output datasets of hard coal-rock were constructed by means of cutting experiments and digital simulation, and then the digital volume of hard coal-rock was constructed by fitting the input and output datasets. The mechanical properties of different coal-rock have not been deeply studied to construct a more accurate digital volume of coal-rock. Further research will be conducted in the future. Thanks to the suggestions of the review experts, this article has significant guiding significance for the research of this paper and has been cited in the paper. (The references in the paper have been modified and marked in highlighted font. As shown below:)

**Modified part:**

[1] Li Q, Liu S, Gao M. Multiobjective optimization of roadheader shovel-plate parameters using gray weight and particle swarm optimization. IEEE Access. 2022 Sep 26;10:104555-66. <https://doi.org/10.1109/ACCESS.2022.3210165>.

[2] Wang S, Wang D, Ma A, et al. Intelligent Robust Control of Roadheader Based on Disturbance Observer. Actuators 2025, 14(1):36. <https://doi.org/10.3390/act14010036>.

[4] Yang W, Zhang X, Ma H, et al. Geometrically driven underground camera modeling and calibration with coplanarity constraints for a boom-type roadheader. IEEE Transactions on Industrial Electronics. 2020 Aug 25;68(9):8919-29. <https://doi.org/10.1109/TIE.2020.3018072>.

[6] Lin, H, Liu, W, Zhang, D, et al. Study on the degradation mechanism of mechanical properties of red sandstone under static and dynamic loading after different high temperatures. Sci Rep 15, 11611 (2025). <https://doi.org/10.1038/s41598-025-93969-4>.

[7] Wang L, Xue Y, Liu J, et al. Mechanical behavior and fracture mechanism of high-temperature granite cooled with liquid nitrogen for geothermal reservoir applications. Physics of Fluids. 2025 Feb 1;37(2). <https://doi.org/10.1063/5.0253668>.

[17] Dong Z, Zhang X, Yang W, et al. Automatic Cutting Speed Control System of Boom-Type Roadheader Based on Geological Strength Index. Minerals. 2022; 12(12):1582. <https://doi.org/10.3390/min12121582>

[18] Tian, Jie, Suyu Wang, Miao Wu. Kinematic models and simulations for trajectory planning in the cutting of Spatially-Arbitrary crosssections by a robotic roadheader. Tunnelling and Underground Space Technology 78 (2018): 115-123. <https://doi.org/10.1016/j.tust.2018.03.032>.

[24] Cheluszka P, Remiorz E, Rostami J. The Use of a Roadheader Simulator in Research of Dynamics and Energy-Consumption of Excavating Underground Roadways and Tunnels. Energies. 2022; 15(18):6673. <https://doi.org/10.3390/en15186673>.

**Question 5:** The entire text contains numerous language errors, such as "100sm" and "DDGP". Please carefully check the entire text and make necessary revisions.

**Response 5: Thank you for pointing out the mistakes.**

We are very sorry for the errors in the paper. We have corrected the mistakes regarding units and words in the paper, and made language modifications and format checks on the entire paper. We sincerely thank you for your strict supervision, which has helped us enhance the professionalism of the paper.

**Question 6:** In the conclusion, avoid restating methodological details and instead focus on highlighting innovative aspects and future research directions.

**Response 6: Thank you for your valuable suggestions.**

The conclusion is restated in the paper.（The conclusion in the paper has been modified and marked in highlighted font. As shown below:）

**Modified part:**

This study has successfully developed a virtual-real fusion testing system for boom-type roadheader cutting modules, demonstrating three core capabilities:

(1) Aiming at the problems of high experimental research cost and safety risk of boom-type roadheaders, a virtual-real fusion experiment system based on chain-type digital mapping bodies is proposed. This system is applicable to virtual experiments, physical entity experiments and virtual-real fusion experiments of the cutting part of the boom-type roadheaders. This system takes Virtual-Dominant Entity Calibration as its functional core and is capable of constructing digital models of coal-rock, simulating mechanical properties of structures, and conducting virtual-real fusion experiments. Subsequently, the DDPG algorithm was adopted to conduct optimization experiments on the control strategy of the cutting part, verifying the feasibility of the system.

(2) Based on deep learning, coal-rock cutting experiments, and numerical simulation, a discretized rectangular unitization recognition method for coal-rock is proposed, and a digital twin of coal-rock for the load of the cutting teeth is constructed. This method can construct digital coal-rock, provide mechanical load input for virtual cutting experiments of the roadheader, realize digital simulation of the complex coal-rock cutting process by the cutting head, and reduce the difficulty and cost of fabricating large pieces of coal-rock in cutting experiments.

(3) Based on coordinate space transformation and ROM technology, a chained digital twin model is proposed. Through data transmission and coordinate transmissions, the pose and mechanical state changes of the cutting part of the boom-type roadheader can be simulated. The overall digital mapping of complex assemblies has been achieved, providing a basic platform for virtual experiments.

The next step is to improve the coal-rock digital mass by studying the mechanical properties, degradation mechanisms, and fracture mechanisms of the surrounding rock to increase accuracy, applying this system to other tunnel boring machines or mining equipment to form a systematic framework, to solve problems of large-scale mechanical testing, and to combine advanced technologies such as artificial intelligence and big data to further enhance the intelligence level of the system.

**Reviewer 3:**

**Question 1:** This paper proposes a virtual-reality fusion experimental system for the cutting part of a cantilevered roadheader, which has important innovations in reducing the difficulty of the experiment and improving the adaptability of the cutting. However, it is recommended to further clarify the actual application scenarios and potential socio-economic value of the system to highlight the innovation and practical contribution of its research.

**Response 1: Thank you for your valuable suggestions.**

The introduction in the paper has been modified and supplemented. The main function of this system is to integrate the experiment prototype, theoretical model and virtual simulation together to construct a virtue-real integrated boom-type roadheader experiment system, which has the function of simulating the cutting experiment of the cutting part of the boom-type roadheader and reduces the experiment difficulty. And it can solve the data acquisition problem during the experiment process of the boom-type roadheader through virtual sensors, providing a basic platform for the research of the boom-type roadheader. The experiment model of "virtual dominance - physical calibration" can reduce the number of coal-rock and sensors on the premise of ensuring the reliability of the experiment. The experiment framework is as follows:

**Fig. a.** The compare experiment process framework

Both the validation experiment and the comparison experiment have less requirements on the number of experimental groups, so the virtual experiment and the physical experiment are carried out simultaneously. Taking the verification experiment as an example, the process framework is shown in Fig. a. First of all, the conclusion to be verified is determined, and then the physical environment and virtual environment are built based on the physical environment, and the virtual experiment and physical experiment are carried out at the same time. If the result of the physical experiment and the virtual experiment is the same, the result is judged to be correct or not, and the experiment conclusion is drawn. If the result of the physical experiment is inconsistent with that of the virtual experiment, then the virtual-real experiment will be conducted. If the reslut of the virtual-real experiment is the same as that of the physical experiment, the virtual experiment environment will be modified and the virtual experiment will be conducted again. If the conclusion of the virtual experiment is the same as that of the virtual experiment, the physical environment is corrected and the physical experiment is re-conducted. Finally, the physical experiment and the virtual-real experiment came to the same conclusion.

**Fig. b.** The optimize experiment process framework

Both the optimization experiment and the exploration experiment require a large number of experimental groups for comparative analysis, so the experiment process is based on the virtual experiment and supplemented by the physical experiment. Take the optimization experiment as shown in Fig. b, first determine the optimization objective, then build a virtual environment, and conduct several virtual experiments until the optimization objective is reached. Then build a physical environment for physical experiment verification and conduct virtual-real experiment verification. If both the physical experiment and virtual-real experiment reach the optimization goal, the experiment will be completed; If neither of them reach the optimization goal, the virtual environment will be modified and start again. If the physical experiment achieves the optimization goal and the virtual-real experiment does not achieve the optimization goal, the virtual environment will be modified; if the physical experiment does not achieve the optimization goal but the virtual-real experiment achieves the optimization goal, the physical experiment environment will be modified and the physical experiment will be conducted again.

(The introduction part in the paper has been modified and marked in highlighted font. As shown below, the experimental framework was not added to the paper)

**Modified part:**

The boom-type roadheader is a tunneling machine designed for rapid excavation in underground coal mine tunnels. Accurate measurement and control of its operational posture, along with the development of intelligent cutting systems and mechanical property optimization, are critical for enhancing tunneling safety and efficiency [1,2]. For instance, intelligent control of rotational and swing speeds during excavation maintains consistent cutting torque and structural stress, thereby minimizing equipment vibration and wear under variable coal-rock conditions [3]. The integration of image processing and deep learning further enhances system intelligence while reducing operational safety risks [4]. Traditional research methods relying on full-scale physical prototypes face limitations including prolonged development cycles, high costs, and challenges in replicating extreme operating conditions. Studies involving pose recognition and adaptive control which depend on sensor data and simulations confront additional hurdles: adverse environmental factors at tunnel faces, difficulties in acquiring real-time coal-rock properties and equipment status data, and stringent safety protocols collectively escalate experimental durations, costs, and risks [5-7]. Overcoming these cost and efficiency barriers remains a pivotal challenge in advancing intelligent roadheader development.

Current methodologies primarily involve physical experiments and numerical simulations. While physical experiments provide reliable data, their implementation involves repetitive tasks, accelerated equipment degradation, and high temporal-economic costs, limiting multi-parameter combinatorial validation. For instance, fabricating coal-rock specimens and deploying sensor networks demand substantial labor and material investments [8]. Data-intensive approaches like deep learning face scalability challenges due to the prohibitive costs of acquiring labeled datasets through full-scale underground or surface cutting experiments [9-11]. Although numerical simulations reduce costs through virtual data generation, two critical limitations persist: (1) accuracy degradation from idealized boundary conditions, and (2) inadequate representation of system-level behaviors when simulating isolated components [12]. System-scale simulations further require complex coupling across multiple software platforms, compounded by the lack of real-time cross-validation between numerical and physical domains [13-16]. This decoupling prevents dynamic error correction, necessitating post-hoc experimental verification of simulation datasets.

Current experimental approaches predominantly employ bench-scale or reduced-dimension prototypes to mitigate the complexity of boom-type roadheader testing. Existing platforms simulate operational states through cutting head pose recognition, control system emulation, and concrete cutting modules. However, material-based simulations (e.g., concrete/gypsum-coal mixtures) lack real-time tunability of mechanical properties, while requiring extensive manual calibration to approximate coal-rock behaviors [17]. Although theoretical modeling and virtual simulations partially alleviate these issues, physical validation remains mandatory [18]. Integrating physical prototypes with theoretical models and virtual simulations into a unified virtual-real testing system could systematically mitigate experimental complexity. Specifically, virtual sensors enable automated data acquisition during testing cycles, circumventing the need for intrusive physical instrumentation. This integration establishes a foundational platform for advanced roadheader research with enhanced self-sensing capabilities.

Digital twin technology represents a prominent research field in virtual-real system integration. This technology enables real-time synchronization between physical entities and their virtual counterparts through bidirectional data exchange [19-21]. Despite these advances, current implementations predominantly focus on pose monitoring and control of boom-type roadheaders, while neglecting integrated mechanical simulation and experimental validation [22-24]. Deep learning exhibits exceptional nonlinear regression capabilities, allowing it to approximate nonlinear interactions between boundary constraints and structural response fields when trained on numerical datasets [25,26]. Such models facilitate the generation of reduced-order simulations and digital twin mappings, thereby accelerating mechanical analysis [27-30]. By coupling physical prototypes with their digital twins, a hybrid experimental framework emerges, unifying virtual and physical testing modalities under a single architecture.

This study aims to develop a cutting module experimental system for boom-type roadheaders, establishing a virtual-dominant physical calibration paradigm to reduce experimental costs, enhance dataset accuracy, and improve system intelligence. The proposed system integrates five core functionalities:

**Question 2:** This paper introduces the construction process of the system in detail, including key technologies such as coal-rock discretization, digital mapping body construction, and virtual-reality system fusion. However, it is recommended to add more technical implementation details, such as the implementation steps, parameter settings, and code implementation of specific algorithms, so that readers can better understand and reproduce the research content.

**Response 2: Thank you for your valuable suggestions.**

The digital volume of coal-rock and the digital mapping volume of the cutting part are the key parts in the virtual-real fusion experimental framework. The core of their construction is to deploy the model fitted by deep learning to C++. The deployment process is as follows: Firstly, the fitting model is read through C++. Then, the dynamic link library is constructed and the dynamic link library file is embedded in the Unreal Engine. Finally, the blueprint files of each model are created by referencing the dynamic link library. The virtual experiment system in the virtual-real fusion experimental framework is built through the blueprints of each model and data transmission, and the physical experiment system is connected through the communication protocol. The construction of the digital body of coal-rock, the digital mapping body of the cutting part and the experimental framework of virtual-real fusion has been modified in the paper. (Sections such as 2.2, 3.1 and 4.1 in the paper have been modified and marked in highlighted font. As shown below:)

**Modified part:**

(1) Modification content of coal-rock digital volume (The last paragraph of Section 2.2)

After constructing the input and output datasets, data normalization is first applied. The datasets are divided into the training set, the validation set and the test set in a ratio of 7:2:1. Subsequently, the Deep Convolutional Neural Networks (DCNN) is employed for fitting training. The DCNN implementation uses TensorFlow with the following architecture: the number of network layers is set to 7 layers, the activation function adopts ReLU, and the loss functions all adopt mean square deviation functions. The trained model is deployed as a C++ executable module, forming the digital coal-rock volume. Finally, spatial stacking of multiple rectangular element digital twins constructs the complete digital coal-rock model.

(2) Modification content of the digital mapping body in the cutting part (Section 3.1, Paragraph 3)

The attitude and mechanical states of each structural body comprise sensor-derived data and digital twin outputs. Among them, the sensor data are obtained from the displacement sensor and pressure sensor from the translational cylinder, the displacement sensor and pressure sensor from the rotary cylinder, the displacement sensor and pressure sensor from the swing arm cylinder, and the torque and speed sensor from the cutting head. Non-sensor data primarily consist the mechanical data and pose correction data of each structure, derived via component-level digital twins. To ensure output accuracy, input-output datasets must encompass diverse operating conditions. The input data set should cover all factors affecting outputs, as shown in Fig. 5. Numerical simulations under various working conditions are conducted by changing the input parameters to cover as many possible working conditions as possible. Mechanical states and pose corrections extracted from these simulations construct the datasets. Deep learning establishes the mapping between input parameters and output responses, enabling real-time prediction of the boom-type roadheader's mechanical states. This approach achieves real-time numerical simulation capabilities, providing stress and deformation data at grid nodes. The digital simulation employs the LS-DYNA solver, the solid adopts the Lagrangian, the oil adopts the Arbitrary Lagrangian Eulerian(ALE), and the joint adopts the Kinematic pair connection.

(3) Modification contents of the virtual-real fusion experiment system (Section 4.1, Paragraph 1)

The whole system is composed of the physical experiment system of the cutting part of the boom-type roadheader and the virtual experiment system. As shown in Fig. 8. The physical experiment system includes the mechanical structure, the hydraulic power system and the electrical control system; The virtual experiment system consists of key structural components digital twin, communication system and visual operating system. The virtual-real fusion experiment system is built with a visual operating system powered by UE5. The communication system includes the S7 communication protocol and the mysql communication protocol. The digital coal-rock model and component-level digital twins are embedded via C++ deployment modules. Firstly, the deployment blueprint is encapsulated. Next, grid node coordinates and temporal sequence data are extracted and fed into the pre-programmed grid for rendering, where material coloration is governed by stress and deformation data. Finally, input-output variables are constructed for data transmission within the chain-structured digital twin framework, and spatial coordinates of each component are regulated via kinematic equations to complete the virtual boom-type roadheader cutting module.

**Question 3:** This paper verifies the accuracy of the virtual-reality fusion experimental system through experiments and demonstrates its effectiveness in reducing the stress of the cutting part. However, it is recommended to further analyze the stability and reliability of the experimental data, such as conducting multiple repeated experiments and using different coal-rock samples for experiments, so as to enhance the persuasiveness of the experimental conclusions.

**Response 3: Thank you for your valuable suggestions.**

Thank you to the expert for the suggestion of increasing the experiments. Although this system can reduce the time and economic cost of the coal-rock cutting experiments, the coal-rock in the verification experiments need to be made into physical coal-rock. Since the coal-rock produced last time has been used up, making a new piece of coal-rock for system verification requires a long preparation time and economic cost. Therefore, verification experiments of different coal-rock samples could not be carried out. We are very grateful to the experts for their research opinions on the mechanical properties of rocks, which have provided guidance for the optimization of this system. Subsequently, we will conduct research on the mechanical properties of coal-rocks of different natures, improve the data fitting method, construct a coal-rock database system, and enhance the system functions.

**Question 4:** The virtual-reality fusion experimental system performs well in real-time and synchronization, but it is recommended to further explore the scalability and maintainability of the system. For example, how to quickly adapt to different types of tunnel boring machines, how to conveniently upgrade and maintain the system, etc. In addition, it is possible to consider introducing more optimization algorithms to improve the overall performance of the system.

**Response 4: Thank you for your valuable suggestions.**

We are very grateful to the expert for their suggestions on system expansion. Currently, we are improving the virtual-real fusion experiment system of the tunnel boring machine, mainly by using the Unreal Engine plugin function to modularize the system design. Each structural digital mapping body is set as an independent model. By changing the deployment model and modifying the transferred parameters, it can be combined with other tunneling equipment and mechanical equipment. At present, this framework has completed the construction of the virtual experiment system for hydraulic supports, the virtual experiment system for hydraulic support test benches, and the virtual experiment system for fully mechanized mining equipment, as shown in the following figure. However, we have not yet conducted in-depth research on the suggestions regarding maintainability and algorithms put forward by the experts. We are grateful for the experts' suggestions and will conduct in-depth research and improvements in the future. (This part is not reflected in this paper)

（a）Hydraulic support	（b）Hydraulic support testing machine

（c）Comprehensive mining equipment test system

**Fig. c.** Virtual experiment system for other equipment

**Question 5:** The abstract of the article needs to be revised, and the importance of the article should be highlighted. There are insufficient references, so more references need to be supplemented. There are too few references, which need to be supplemented to 30. The background and mechanism are not introduced clearly.

Mechanical behavior and fracture mechanism of high-temperature granite cooled with liquid nitrogen for geothermal reservoir applications. Physics of Fluids 2025; 37 (2): 026616. https://doi.org/10.1063/5.0253668

**Response 5: Thank you for your valuable suggestions.**

The abstract part has been rewritten in the paper, the introduction has been modified and supplemented, and references have been added. Thank you for your guidance on the mechanical properties of rocks, which has pointed out the direction for improving the accuracy of coal-rock digital bodies in the future. (The modification of the introduction has been described in the above issue. Here, only the modified parts of the references are marked. The modified references are marked in highlighted font in the paper. As shown below:)

**Modified part:**

(1) Abstract

Aiming at the problems of high safety risks, economic costs, and inefficiency in experimental research on boom-type roadheaders, this study proposes a virtual-real fusion system for the cutting module. This system incorporates functions including digital modeling of coal-rock, simulation of mechanical properties of the cutting unit, and integration of virtual and physical experiments. To address the challenge of obtaining cutting tooth loads at coal-rock interfaces, a discretized digital coal-rock volume construction method is proposed. For rapid mechanical performance simulation of the cutting unit, a chain-type digital mapping body construction method is developed. Through deep learning, numerical simulation, and digital twin technologies, a virtual-real fusion platform was established, enabling virtual experiments to dominate the calibration of physical experiments. The system is capable of simulating pose variation and vibration trends of the entire machine during cutting. The minimum average error for stress at the cylinder base is 4.44 MPa, with a virtual-real system connection period under 100 ms. Based on this system, a reinforcement learning training environment was developed. Using Deep Deterministic Policy Gradient (DDPG), the control of the cutting unit was optimized to achieve low-stress state cutting, verifying the system’s feasibility.

(2) References

[1] Li Q, Liu S, Gao M. Multiobjective optimization of roadheader shovel-plate parameters using gray weight and particle swarm optimization. IEEE Access. 2022 Sep 26;10:104555-66. <https://doi.org/10.1109/ACCESS.2022.3210165>.

[2] Wang S, Wang D, Ma A, et al. Intelligent Robust Control of Roadheader Based on Disturbance Observer. Actuators 2025, 14(1):36. <https://doi.org/10.3390/act14010036>.

[4] Yang W, Zhang X, Ma H, et al. Geometrically driven underground camera modeling and calibration with coplanarity constraints for a boom-type roadheader. IEEE Transactions on Industrial Electronics. 2020 Aug 25;68(9):8919-29. <https://doi.org/10.1109/TIE.2020.3018072>.

[6] Lin, H, Liu, W, Zhang, D, et al. Study on the degradation mechanism of mechanical properties of red sandstone under static and dynamic loading after different high temperatures. Sci Rep 15, 11611 (2025). <https://doi.org/10.1038/s41598-025-93969-4>.

[7] Wang L, Xue Y, Liu J, et al. Mechanical behavior and fracture mechanism of high-temperature granite cooled with liquid nitrogen for geothermal reservoir applications. Physics of Fluids. 2025; 37 (2): 026616. <https://doi.org/10.1063/5.0253668>.

[17] Dong Z, Zhang X, Yang W, et al. Automatic Cutting Speed Control System of Boom-Type Roadheader Based on Geological Strength Index. Minerals. 2022; 12(12):1582. <https://doi.org/10.3390/min12121582>

[18] Tian, Jie, Suyu Wang, Miao Wu. Kinematic models and simulations for trajectory planning in the cutting of Spatially-Arbitrary crosssections by a robotic roadheader. Tunnelling and Underground Space Technology 78 (2018): 115-123. <https://doi.org/10.1016/j.tust.2018.03.032>.

[24] Cheluszka P, Remiorz E, Rostami J. The Use of a Roadheader Simulator in Research of Dynamics and Energy-Consumption of Excavating Underground Roadways and Tunnels. Energies. 2022; 15(18):6673. <https://doi.org/10.3390/en15186673>.

**Question 6:** The virtual-real fusion experimental system proposed in the article has broad application prospects, especially in the fields of coal mining and machinery manufacturing. It is recommended that the author further explore the application potential and promotion strategy of the system, and clarify the future research direction. For example, we can study how to apply the system to other types of tunnel boring machines or mining equipment, or explore how to combine advanced technologies such as artificial intelligence and big data to further improve the intelligence level of the system.

**Response 6: Thank you for your valuable suggestions.**

Thank you for your recognition of the virtual-real fusion experimental system and for providing future research directions. We will integrate your guidance and suggestions, as well as cutting-edge research achievements in rock mechanical properties, artificial intelligence and big data, to improve the framework of the virtual-real fusion test system, making it a systematic framework capable of conducting virtual-real fusion tests on various complex mechanical equipment with high precision, and providing a high-efficiency and low-cost test platform for all experts and scholars. The conclusion has been modified in the paper. (The conclusion in the paper has been modified and marked in highlighted font. As shown below:)

**Modified part:**

This study has successfully developed a virtual-real fusion experimental system for boom-type roadheader cutting modules, demonstrating three core capabilities:

(1) Aiming at the problems of high experimental research cost and safety risk of boom-type roadheaders, a virtual-real fusion experiment system based on chain-type digital mapping bodies is proposed. This system is applicable to virtual experiments, physical entity experiments and virtual-real fusion experiments of the cutting part of the boom-type roadheaders. This system takes Virtual-Dominant Entity Calibration as its functional core and is capable of constructing digital models of coal-rock, simulating mechanical properties of structures, and conducting virtual-real fusion experiments. Subsequently, the DDPG algorithm was adopted to conduct optimization experiments on the control strategy of the cutting part, verifying the feasibility of the system.

(2) Based on deep learning, coal-rock cutting experiments, and numerical simulation, a discretized rectangular unitization recognition method for coal-rock is proposed, and a digital twin of coal-rock for the load of the cutting teeth is constructed. This method can construct digital coal-rock, provide mechanical load input for virtual cutting experiments of the roadheader, realize digital simulation of the complex coal-rock cutting process by the cutting head, and reduce the difficulty and cost of fabricating large pieces of coal-rock in cutting experiments.

(3) Based on coordinate space transformation and ROM technology, a chained digital twin model is proposed. Through data transmission and coordinate transmissions, the pose and mechanical state changes of the cutting part of the boom-type roadheader can be simulated. The overall digital mapping of complex assemblies has been achieved, providing a basic platform for virtual experiments.

The next step is to improve the coal-rock digital mass by studying the mechanical properties, degradation mechanisms, and fracture mechanisms of the surrounding rock to increase accuracy, applying this system to other tunnel boring machines or mining equipment to form a systematic framework, to solve problems of large-scale mechanical testing, and to combine advanced technologies such as artificial intelligence and big data to further enhance the intelligence level of the system.
