# Supplementary material for: Research on a virtual-real fusion experimental system for the cutting part of a boom-type roadheader
Source: PLoS One. 2025 Aug 18;20(8):e0330291. doi: 10.1371/journal.pone.0330291 (PMC12360573; doi:10.1371/journal.pone.0330291)
Supplement: Attachment — Submitted filename: response_letter_auresp_2.doc [file pone.0330291.s007.doc]

**List of Responses**

Dear editors and reviewers:

We have carefully studied your valuable opinions on our manuscript "Research on virtual-real fusion experiment system of the cutting part of the boom-type roadheader". Thank you for giving us the opportunity to make revisions.

We highly value your insightful comments on the previous version. In response, we have carefully processed each comment as follows. All the modified parts are highlighted in the original draft. We sincerely hope that our revisions meet your expectations.

**Reviewer 2:**

**Question 1:** Aiming at the problems of high safety risks, economic costs, and inefficiency in experimental research on boom-type roadheaders, this study proposes a virtual-real fusion system for the cutting module. This system incorporates functions including digital modeling of coal-rock, simulation of echanical properties of the cutting unit, and integration of virtual and physical experiments.

**Response 1:** Thank you very much for your valuable comments and your affirmation of the research significance of our manuscript. We sincerely appreciate the time and effort you dedicated to reviewing our work. Your recognition serves as a significant encouragement to us, motivating us to strive for excellence in our scientific research and produce more high-quality academic outcomes. Once again, we express our deepest gratitude to you.

**Reviewer 4:**

**Question 1:** The introduction focuses primarily on coal mine applications. To strengthen the rationale, explicitly reference non-coal applications of boom-type roadheaders (e.g., civil tunnel construction, hydropower caverns, or urban underground infrastructure (https://doi.org/10.1016/j.scs.2022.104369; https://doi.org/10.1016/j.tust.2023.105382)) and cite recent studies demonstrating their use in diverse geotechnical settings (e.g., hard rock tunneling in metro projects). This would better justify the system’s universal value beyond coal-centric scenarios.

**Response 1:** Thank you for your valuable suggestions. We agree that broadening the application context strengthens the rationale. Following your recommendation, the Introduction section has been revised to explicitly reference non-coal mining applications of boom-type roadheaders, such as civil tunnel construction, hydropower caverns, and urban underground infrastructure. To support this, we have cited recent studies demonstrating their use in diverse geotechnical settings, specifically including Liu et al. (2023) on dust suppression in a metro tunnel (*Sustainable Cities and Society)* and Liu et al. (2023) on dust diffusion during partition excavation (*Tunnelling and Underground Space Technology*). These additions (highlighted in the revised manuscript) better justify the universal value of the proposed system beyond coal-centric scenarios. The corresponding references have also been added to the References section (also highlighted).

**Question 2:** Expand testing beyond hard coal-rock at fixed RPM (90 rpm). Include diverse coal-rock strengths (soft/heterogeneous strata) and dynamic operational scenarios (variable cutting speeds, intermittent loads) to demonstrate system robustness. Quantify the impact of 100ms latency on real-time control stability.

**Response 2:** Thank you for your valuable suggestions regarding expanding the testing scope. We acknowledge the importance of evaluating system robustness under diverse conditions.

We appreciate the suggestion to conduct additional tests with soft/heterogeneous strata and variable operational parameters. However, due to significant resource constraints (specifically, the substantial time and economic cost required to fabricate large new coal-rock samples of the required types and sizes after the depletion of the initial hard coal-rock samples), comprehensive physical verification across all suggested scenarios was not feasible within the scope of this revision phase.

The primary objective of this initial research stage was to validate the feasibility of the core virtual-real fusion concept. Selecting hard coal-rock (at a fixed 90 rpm) represented a challenging case for system validation, as it induces larger amplitude structural stress deformations, posing a stricter test for system accuracy. Testing under milder conditions (softer rock, lower RPM) was expected to yield smaller prediction errors but was deemed less critical for the initial feasibility demonstration and constrained by resources.

We sincerely appreciate you highlighting the need to quantify the impact of the 100ms latency on real-time control stability. Our analysis indicates that the primary effect of this latency is potential distortion in the system's predicted data when the cutting head rotational speed exceeds 90 rpm. Crucially, its impact on the actual pose control of the cutting unit is relatively minor. This is because the hydraulic cylinders controlling the swing and rotation operate at slower speeds: within the 100ms interval, the maximum displacement change of these cylinders (under pressures of 7-8 MPa) is only 0.57 mm. Therefore, we conclude that the 100ms latency has a negligible effect on the stability of the pose control within the virtual-real fusion experimental system for the roadheader cutting unit.

We are grateful for your insightful comments on system performance testing, which provide essential guidance for future optimization. Future work will explicitly focus on: (1) Conducting mechanical performance studies on different coal-rock types under varying cutting speeds; (2) Improving data fitting methods; (3) Building a comprehensive coal-rock database system; and (4) Enhancing overall system functionality and robustness based on these findings.

**Question 3:** Clarify the deep learning architecture (e.g., layer types, node counts, optimization algorithms) and validation metrics (e.g., RMSE, R²) used to derive the damage coefficient (Eq. 4). Justify why a 3×3×3 boundary matrix (Eq. 5) suffices for spatial heterogeneity modeling.

**Response 3:** Thank you for your valuable suggestions seeking clarification on the deep learning model and spatial modeling rationale.

I apologize for the unclear description of the damage coefficient of the cut coal-rock cubic units in the paper. To obtain the external load conditions of the boom-type roadheader and simulate its mechanical state, it is necessary to determine the mechanical state of the cutting picks. First, the coal-rock mass is discretized into multiple cubic units (). For each cubic coal-rock unit , a mapping relationship $f_{io}\text{：}\left[i\right]\to\left[o\right]$ is established to couple coal-rock information with pick data, where $\left[i\right]$ represents parameters influencing the pick's mechanical state and $\left[o\right]$ denotes the pick's mechanical state information. Multiple identical or different units can then form larger coal-rock blocks to simulate the cutting process of bulk coal-rock. Since particle spallation occurs during coal-rock cutting, the impact of coal-rock fragmentation on pick forces must be considered. During the cutting of each cubic unit, the pick's mechanical state is affected by the damage status of the surrounding rock mass. Therefore, parameter $\left[i\right]$ includes the damage coefficient of the coal-rock surrounding the cubic unit, specifically the 3×3×3 spatial information matrix . As hard coal-rock is less prone to large-scale fracturing, non-adjacent coal-rock units have minimal impact on the cutting process of . Consequently, the damage degree of non-adjacent coal-rock units was not included in the input parameters $\left[i\right]$.

In the datasets for deep learning-based fitting of$f_{io}\text{：}\left[i\right]\to\left[o\right]$， the damage coefficient of rectangular coal-rock units serves as both input and output data. The fitting model corresponds to the training model for $f_{io}\text{：}\left[i\right]\to\left[o\right]$，with specific details as follows: After constructing the input and output datasets, data normalization is first applied. The datasets are divided into training, validation, and test sets in a 7:2:1 ratio. Subsequently, Deep Convolutional Neural Networks (DCNN) are employed for fitting training. The DCNN is implemented using TensorFlow with the following architecture: 7 network layers, ReLU activation function, and mean square deviation loss functions. The trained model is deployed as a C++ executable module to form the digital coal-rock volume. Finally, spatial stacking of multiple rectangular element digital twins constructs the complete digital coal-rock model.

To address the requirement that the damage coefficient functions as both input and output data, the datasets construction process records the pre-cutting damage coefficient as input data and the post-cutting damage coefficient as output data. During the simulated cutting process, the initial uncut value is set to 1, with all values in matrix initialized to 1. Subsequent cutting process prediction is achieved through chain data transmission.

Section 2.1 has been revised accordingly and is highlighted.

**Question 4:** Explicitly compare resource requirements (time, materials, labor) between traditional physical experiments and the proposed virtual-dominant approach (e.g., 300 virtual + 10 physical DDPG runs). Estimate savings in coal-rock fabrication, sensor deployment, and downtime to highlight scalability.

**Response 4:** Thank you for your valuable suggestion to quantify the resource savings. We explicitly compare the requirements below, using the DDPG algorithm training example (300 virtual + 10 virtual-real fusion runs):

In terms of safety: In conducting reinforcement learning control training for roadheaders using traditional physical experiments, certain safety risks may arise, as exploratory actions could lead to hazardous machine operations and potential accidents. In contrast, the virtual-physical fusion test system enables extensive reinforcement learning training to be performed first in a virtual environment, mitigating accident risks associated with exploratory actions. After the control strategy stabilizes, physical experiments can then be used for correction and refinement.

In terms of resource consumption: Traditional physical experiments would require approximately 52 blocks of 2000×1500×1500mm coal-rock specimens to conduct 310 linear cutting tests. With an estimated workforce of 3-5 personnel for specimen preparation, 2 sets of casting molds, and 1 operator for cutting tests, this approach would take roughly 2-3 years. Furthermore, it can only capture structural stress at strain sensor locations, making it nearly infeasible for deep reinforcement learning applications.

In comparison, the virtual-physical fusion system—utilizing 300 virtual cutting tests and 10 virtual-physical fusion training cycles—relies on computer-based numerical simulations. Both coal-rock cutting and structural mechanics simulations employ small-scale models, which can be parallelized across multiple computers, significantly reducing time consumption. The construction of the fusion system in this study required approximately 3 months. For datasets development, 10 blocks of 300×300×300mm coal-rock specimens were used, while the 10 virtual-physical fusion tests consumed 2 blocks of 2000×1500×1500mm specimens. Regarding labor costs: 3-5 personnel were required for preparing the 2 large specimens, while all other tasks could be completed by a single researcher.

Once established, the virtual-physical fusion test system allows unlimited virtual experiments without accident risks. Although additional costs are incurred for fusion experiments, structural stress and deformation data can be acquired at any location on the cutting unit.

The last paragraph of Section 5.3 in the paper has been revised and is highlighted.

**We deeply appreciate the reviewers' expertise and constructive feedback, which has strengthened this work. While resource limitations prevented full implementation of all suggestions (notably expanded physical testing in Q2/Q4), we are actively pursuing these directions in ongoing research to further enhance the system's robustness and applicability.**
